# Lateral forces on circularly polarizable particles near a surface

**DOI:** 10.1038/ncomms9799

**Published:** 2015-11-19

**Authors:** Francisco J. Rodríguez-Fortuño, Nader Engheta, Alejandro Martínez, Anatoly V. Zayats

**Affiliations:** 1 Department of Physics, King's College London, London WC2R 2LS, UK; 2 Department of Electrical and Systems Engineering, University of Pennsylvania, Philadelphia, Pennsylvania 19104, USA; 3 Departamento de Comunicaciones, Nanophotonics Technology Center, Universitat Politècnica de València, 46022 Valencia, Spain

## Abstract

Optical forces allow manipulation of small particles and control of nanophotonic structures with light beams. While some techniques rely on structured light to move particles using field intensity gradients, acting locally, other optical forces can ‘push' particles on a wide area of illumination but only in the direction of light propagation. Here we show that spin–orbit coupling, when the spin of the incident circularly polarized light is converted into lateral electromagnetic momentum, leads to a lateral optical force acting on particles placed above a substrate, associated with a recoil mechanical force. This counterintuitive force acts in a direction in which the illumination has neither a field gradient nor propagation. The force direction is switchable with the polarization of uniform, plane wave illumination, and its magnitude is comparable to other optical forces.

## Abstract (web summary)

Some optical forces can direct particles, but only in the direction of light propagation. Here, the authors show theoretically that when the spin of the incident circularly polarized light is converted into lateral electromagnetic momentum, it leads to a lateral optical force associated with a recoil mechanical force.

## Graphical abstract

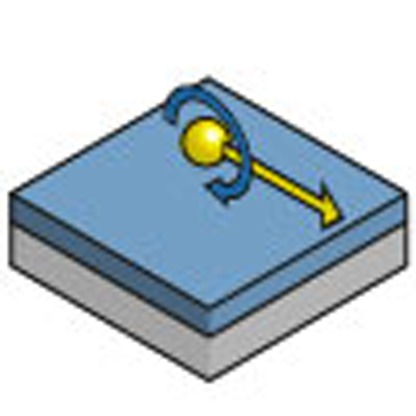

The transfer of momentum from electromagnetic radiation to matter enables the existence of optical forces^1^,^2^, allowing the trapping and manipulation of particles^3^,^4^,^5^,^6^, single cells^7^ and atoms^8^ with important applications such as optical cooling^9^ or lab-on-a-chip microfluidic sorting^10^ amongst many others^11^. There is a wide variety of optical forces, such as radiation pressure force^3^,^4^, exotic metamaterial forces for adhesion or repulsion^12^,^13^,^14^,^15^, and gradient forces in optical tweezers^11^,^16^, waveguides^17^,^18^,^19^ and nanostructures^20^,^21^. Yet, all these examples are either (i) localized to a single focused beam (for example, optical tweezers) or field gradients in the vicinity of a nanostructure, or (ii) allow limited manipulation, with the force acting in the illumination direction (for example, pressure force) or perpendicular to a substrate.

The possibility of a net force that acts simultaneously on several particles at different locations within a wide area (not requiring focusing of a light beam onto the individual objects), and, in addition, is directed laterally (parallel to the substrate and perpendicular to the illumination direction), would enable the mass movement, arrangement and sorting of particles on a substrate or waveguide in a simple way. Such a net total force acting on a particle perpendicular to the direction of plane wave illumination is different from the well-known torque forces exerted on particles illuminated by circular polarization due to the transfer of angular momentum^22^,^23^,^24^,^25^,^26^. Recent theoretical proposals have unveiled lateral optical forces acting on particles near a substrate^27^,^28^, but they rely on the use of chiral particles to break the symmetry. Polarization-dependent lateral forces have been proposed and measured, arising from transverse momentum and spin of incident light in evanescent and structured light fields^29^,^30^,^31^, but these forces act only on larger particles and vanish for scatterers in the dipolar approximation.

In this paper we ask whether net lateral forces, normal to the plane of incidence, can be achieved for arbitrary objects (including non-chiral and centrosymmetric particles, as well as small particles or atoms in the dipole approximation) with far-field, plane wave illumination, acting on an ensemble of objects simultaneously. We demonstrate, analytically and numerically, the presence of such counterintuitive lateral force, which does not exist for linearly polarized dipolar scattering, and which switches its direction for circularly polarized dipoles of opposite handedness, thus providing opportunity to control it uniquely with the polarization of the illuminating light.

## Results

### Origin of the lateral force

**Figure 1:**
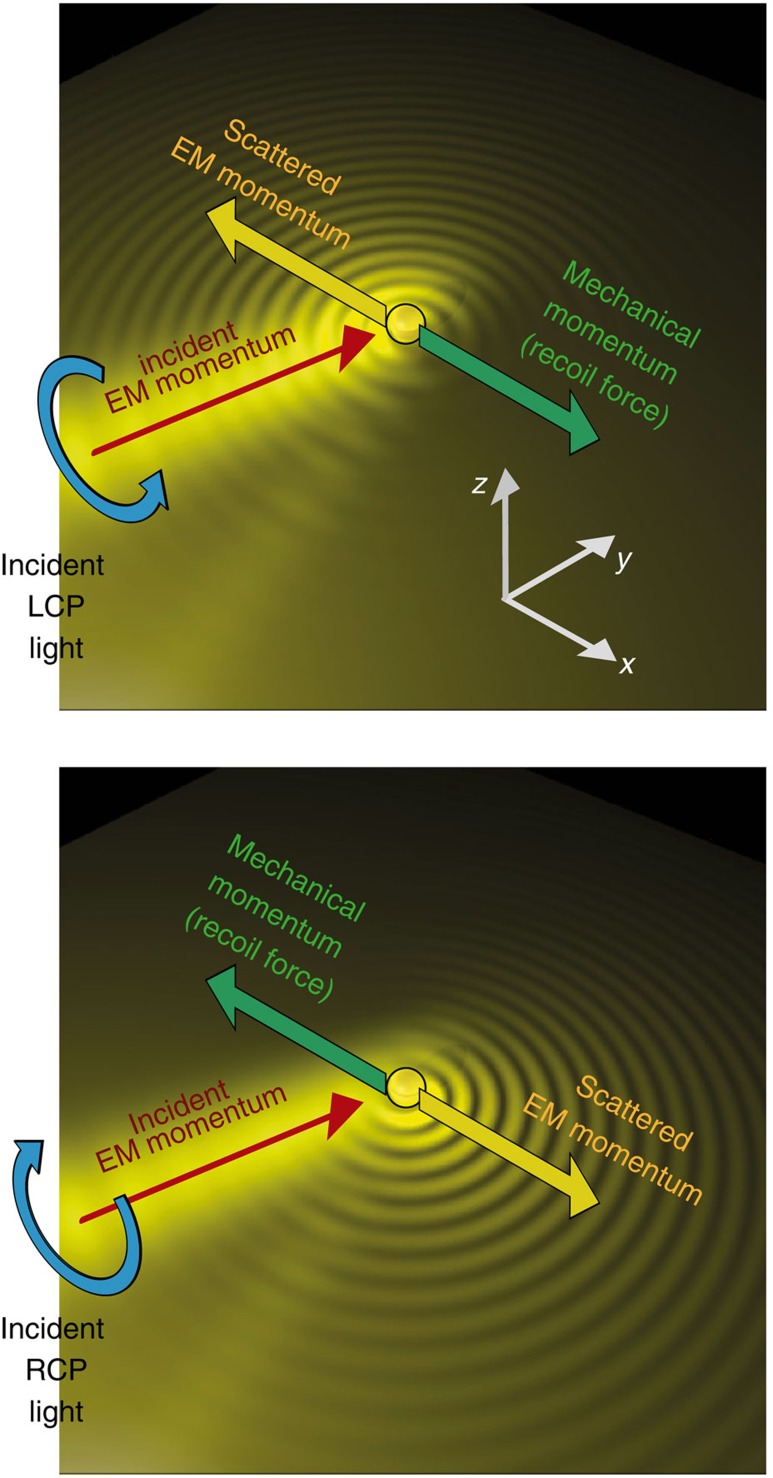
Spin–orbit coupling for polarization-dependent recoil forces. Polarized illumination of a nanoparticle over a surface supporting guided modes results in unidirectional excitation of guided modes and a corresponding polarization-dependent recoil force on the particle. Background image is the intensity distribution of the SPP excited by a circularly polarized dipolar particle, simulated using the Green function approach.

The phenomenon relies on spin–orbit coupling of light mediated by a particle near a surface, caused by the fundamental relation between the transverse spin of the electric field and the evanescent wave propagation direction^32^,^33^,^34^,^35^. It was experimentally shown that by shining circularly polarized light into a nanoparticle placed on a surface^36^,^37^, the dipole-like scattering of the particle excites surface modes on the nearby interface with a strong polarization-dependent unidirectionality (Fig. 1). This is an inherent property of the spin (circular polarization) of the electric near-field^33^, and, therefore, works not only on metallic surfaces but on any structure supporting guided electromagnetic modes^32^,^33^,^34^,^35^,^36^,^37^,^38^,^39^,^40^,^41^,^42^,^43^,^44^,^45^,^46^,^47^,^48^. A non-symmetric scattering is achieved even though the geometry (and the direction of light incidence) is completely symmetric (Fig. 1); the symmetry breaking is achieved by the polarization of the illumination alone. In this case the existence of a lateral force follows directly from considerations of momentum conservation. The illuminating beam carries momentum in the direction of illumination only, with a zero lateral component (in *x* direction). However, the unidirectional scattering of light has non-zero electromagnetic momentum in the *x* direction. Conservation of momentum implies that the particle, by launching the electromagnetic waves unidirectionally, must necessarily be imparted with an equal and opposite mechanical momentum that cancels out the total lateral momentum (similar to the recoil force experienced by atoms on emission of photons^49^). Since the scattering direction is determined by the incident polarization (spin–orbit coupling), the direction of the recoil force can be switched simply by switching the incident polarization from one handedness to another (Fig. 1). Electromagnetic momentum can thus be intentionally excited in a given direction, to harness the mechanical momentum that pushes back the particle in the opposite direction. In the presence of losses, the surface electromagnetic mode will eventually be absorbed by the substrate, and its momentum transferred to it.

### Numerical simulations of illuminated particle

**Figure 2:**
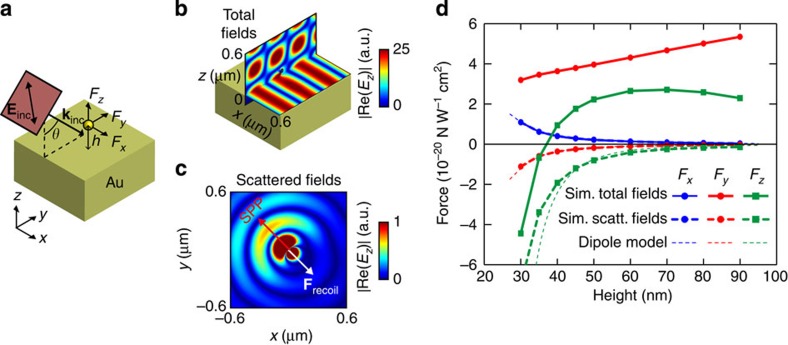
Numerical simulation of illuminated gold nanoparticle near surface. (**a**) Schematics of illumination. (**b**,**c**) Distribution of electric field in a simulation with *h*=45 nm for (**b**) the total electric field (incident+scattered) and (**c**) the scattered electric field. In both cases the depicted *x*–*y* plane is 4 nm above the surface. (**d**) The distance dependence of the optical forces acting on the particle near the metal surface, calculated using Maxwell's stress tensor. Note that the simulated *F*_*x*_ force induced by the scattered field is identical to that induced by the total field, and agrees closely with the dipole model prediction. For the dipole model, equation (1) is used to calculate *F*_*x*_ (and *F*_*y*_ by appropriately reorienting the axes), while *F*_*z*_ was calculated following ref. 15. The dipole moment **p** used for the model was derived (for each height) from the scattered fields. Gold particle diameter is 40 nm, illuminating wavelength is *λ*=520 nm, plane wave incidence 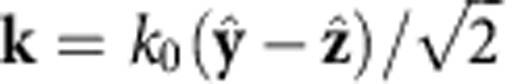 (incidence angle *θ*=45°), incident polarization 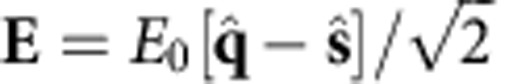, where 
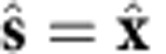
 corresponds to *s*-polarization and 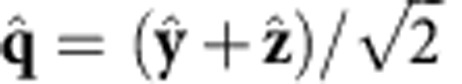 corresponds to *p*-polarization. The lateral force 
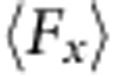
 switches sign if the polarization switches to 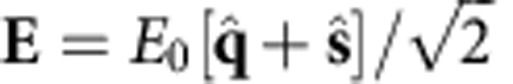 (not shown).

Figure 2 shows a numerical simulation of the force acting on a 40-nm-diameter spherical gold nanoparticle placed near a gold surface as a function of its distance *h* from the surface, under illumination at 45° incident angle with a linearly polarized plane wave (polarization breaks *x*-mirror symmetry), at a wavelength *λ*=520 nm corresponding to the plasmonic resonance of the isolated particle (*ɛ*_Au_=−3.91+2.60*i*). After reflection at the surface, the incident linearly polarized plane wave creates a standing wave that is elliptically polarized at the location of the particle. The fields are computed using CST Microwave Studio, and the force components 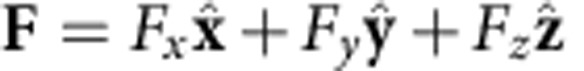 are rigorously calculated from integration of Maxwell's stress tensor in closed surfaces around the particle. Unidirectional mode excitation is observed in the scattered field, and a non-zero lateral force *F*_*x*_ is obtained for a spherically symmetric particle. This contrasts with previous works in which lateral forces are only associated to asymmetric particles^27^, which is only true for incident polarizations that do not break *x*-mirror symmetry. The existence of this lateral force is the main result of this paper, and we see that if the particle is sufficiently close to the surface, it can be of the same order of magnitude as other well-known optical forces such as the pressure force (*F*_*y*_).

To separate various contributions to the force, two scenarios were considered: (i) using the total fields (illuminating plane wave+particle scattering) and (ii) using only the fields scattered by the particle (by subtracting the incident fields from the total fields). In case (i), the force components *F*_*y*_ and *F*_*z*_ include the well-known pressure force and the gradient force caused by the standing wave developed near the metal surface. No lateral force *F*_*x*_ is expected from the illuminating field since there are no gradients and no propagation in the *x* direction. The observed lateral force must therefore arise from the scattering by the particle alone. Indeed, the lateral force *F*_*x*_ induced by the total fields (i) is identical to that induced by the scattered fields alone (ii), supporting this interpretation (Fig. 2d). The scattered fields show unidirectional excitation of surface plasmon waves in the forward and lateral directions, caused by spin–orbit coupling (Fig. 2c). This scattering, usually neglected in force calculations on small particles, is essential to obtain the lateral (*F*_*x*_) and backward (−*F*_*y*_) recoil forces, as shown in the simulations.

### Radiating dipole source model

**Figure 3:**
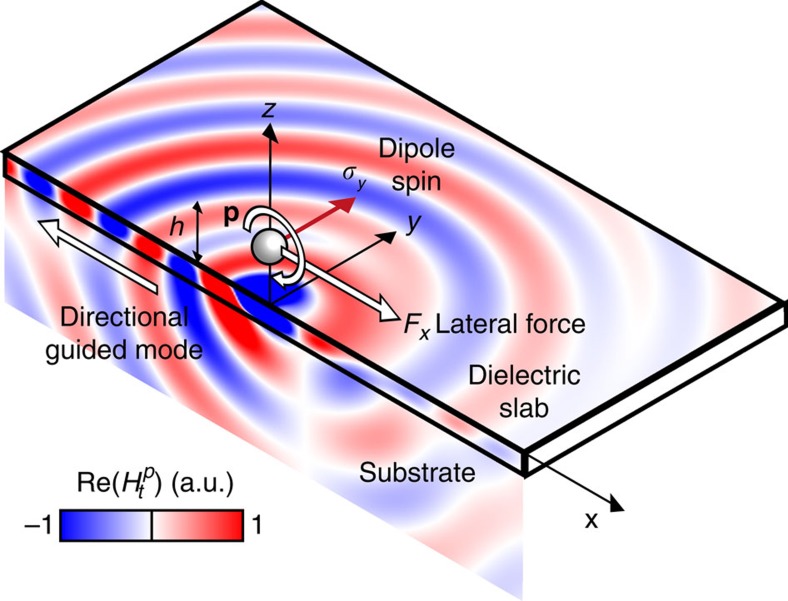
Dipole scattering near a surface. Calculated magnetic field of a circularly polarized dipole at a distance *h* above a dielectric slab. The dipole handedness, the direction of the guided mode excitation in a slab, and the lateral force are indicated by coloured arrows. The parameters of the model correspond to example 1 in the text (*n*_slab_=3.45, *n*_subs_=1.45, *t*_slab_=0.135*λ*).

The scattered fields obtained above are in close agreement to those of a radiating point dipole. This follows from the Rayleigh scattering approximation when the particle is small enough. To understand the origins of this lateral force and provide a simple analytical description, we now consider a point electric dipole with arbitrary polarization **p** placed in free space at a height *h* above a planar surface at *z*=0, as shown in Fig. 3. This generalizes our proposal, because the dipole scenario is not limited to scattering of illuminated particles, but includes driven electromagnetic sources such as emitting quantum dots, and optical, microwave or radio frequency antennas. The nature of a point dipole and the rotational symmetry of a plane surface intuitively suggest that the total time-averaged force acting on the dipole should always be aligned on an axis perpendicular to the surface, either attracting the dipole towards the surface or repelling it away^15^,^50^, being the origin of, for example, the van der Waals and Casimir attractive forces^1^,^51^,^52^. However, we show below that a lateral force exists if the dipole polarization **p** has a spin component parallel to the surface.

From the point of view of the dipole, the surface is completely characterized by its Fresnel reflection coefficients *r*^*p*^(*k*_t_) and *r*^*s*^(*k*_t_) for *p*-polarized and *s*-polarized waves, respectively, where 
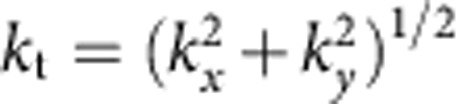
 is the transverse wave vector. We assume a time harmonic polarization of the dipole **p**(*t*)=Re{**p***e*^−*iωt*^}, with angular frequency *ω*=2*πc*_0_/*λ*, , where **p**=[*p*_*x,*_, *p*_*y*_, *p*_*z*_]. We derive the force from first principles, considering the dipole as oscillating equal charges with opposite signs ±*q*, which radiate an electromagnetic field. On interaction with the substrate, the radiated field will be reflected back and exert an electromagnetic force on the charges, in accordance to **F**=*q*(**E**+**v** × **B**). When this equation is applied to the two oscillating charges of a single point dipole and the resulting force is averaged in time^1^,^53^,^54^, it can be written in terms of the electric field only, as 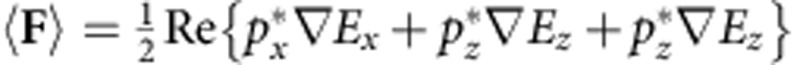, where **** is the gradient operator, evaluated at the location of the dipole. Since the aim of this work is to study the lateral forces (perpendicular to the *z* axis) acting on the dipole, we will focus on the *x* component of the force, without loss of generality, since the *x* axis can be reoriented into any desired direction in the *x*–*y* plane. After substituting the fields of the dipole reflected by the surface into the equation of the force (Supplementary Notes 1 and 6), we arrive at a compact exact equation for the time-averaged lateral force acting on the dipole:


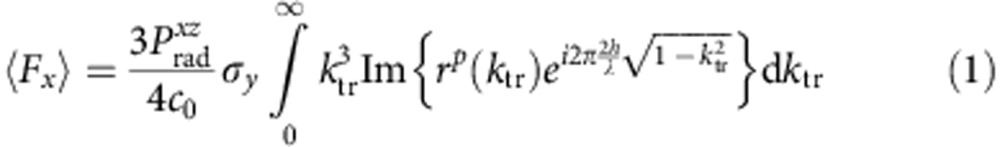


where the integration is performed over the normalized transverse wave-vector variable *k*_tr_=*k*_t_/*k*_0_, *k*_0_=*ω*/*c*_0_, 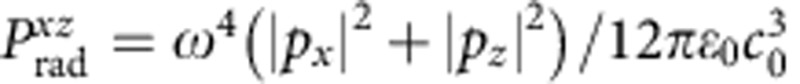 is the power radiated by the *x* and *z* components of the dipole if it was placed in free space, used as an experimentally relevant measure of the amplitude of the dipole excitation, and 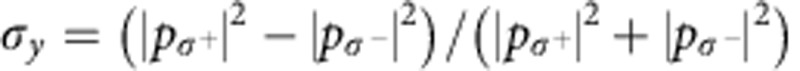 is the polarization spin around the *y* axis (or chirality factor) which equals ±1 for circularly polarized dipoles with opposite rotating sense, and 0 for linearly polarized dipoles (which therefore do not experience any lateral force). The form of *σ*_*y*_ is identical to the third Stokes parameter of a polarized electric field, often used as a measure of the local ‘chirality' or ‘spin' carried by the fields. It is clear from equation (1) that the lateral force is zero in the absence of this spin and is independent of frequency (for a fixed *h*/*λ*). Only the *p*-polarized components of the field appear in equation (1) and, therefore, only transverse magnetic modes excited at the surface will affect the force. Note that the *y* component of the electric dipole does not affect the *x* component of the lateral force, and vice versa.

For added physical insight, it is highly convenient to approximate the force (equation (1)) as three separate contributions (Supplementary Note 2 and Supplementary Fig. 1):


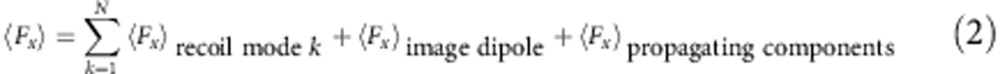


**Figure 4:**
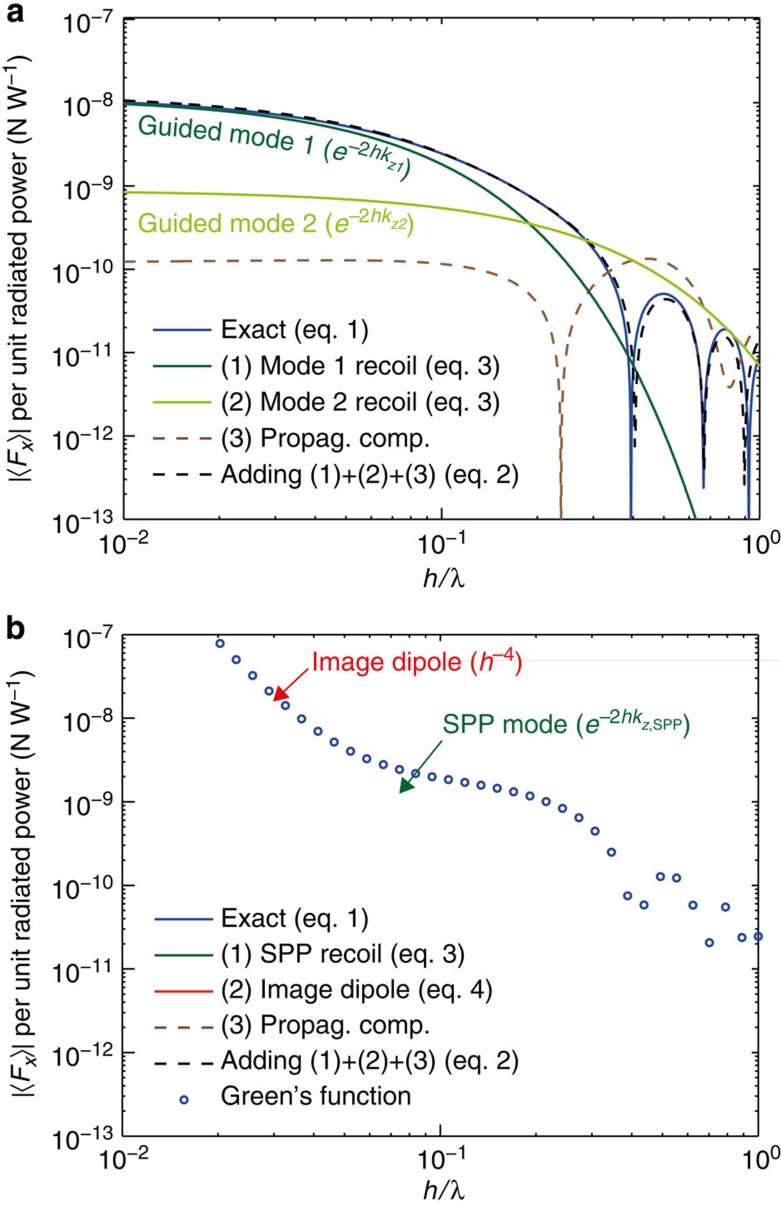
Distance dependence of the time-averaged lateral force. A circularly polarized dipole **p**=[1,0,*i*] above (**a**) a dielectric slab (*n*=3.45) of thickness *t*=0.135*λ* resting on a substrate (*n*=1.45) and (**b**) a gold substrate (*ɛ*_2_=−11.8+1.23*i* at 632.8 nm). The exact result from equation (1) and the different terms in the approximation are shown. Note: for the calculation of *R*_*k*_ we used Δ_1_=0.3*n*_eff,1_, Δ_2_=0.15*n*_eff,2_ and Δ_SPP_=0.1*n*_eff,SPP_. The results of the Green function evaluation are also shown in **b**. Results based on numerical simulations of the same scenarios are shown in Supplementary Note 5.

The accuracy of the approximation (equation (2)) and the distinct behaviour of the different terms is clearly observed in Fig. 4, showing the distance *h* dependence of the total force together with the contribution from the different terms.

The first term is the ‘recoil' force caused by the directional excitation of any transverse magnetic modes supported by the surface, labelled as *k*=1, … , *N*. If the surface is metallic, a single surface plasmon mode exists (*N*=1). A dielectric slab, on the other hand, might support several transverse magnetic guided modes. In the absence of guided modes, such as in a dielectric substrate, *N*=0, and the first term vanishes. It is evident, as depicted schematically in Fig. 3, that the unidirectional excitation of momentum-carrying electromagnetic modes by a spin-carrying dipole implies—in accordance with the conservation of momentum—the existence of a ‘recoil' force acting on the source. This lateral force is directed opposite to the mode excitation and, therefore, parallel to the surface. A simple expression for this term can be deduced by recognizing that any mode will manifest itself in equation (1) as a sharp resonant peak in the reflection coefficient *r*^*p*^(*k*_tr_). We can approximate the imaginary part of *r*^*p*^(*k*_tr_) in equation (1) with a sum of Dirac δ-functions corresponding to the different modes 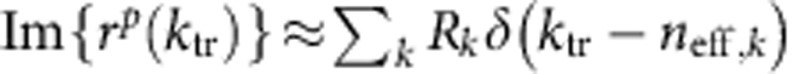, where *n*_eff,*k*_>1 is the effective mode index of the *k*-th mode, and 
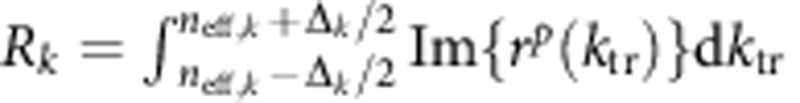
 is a measure of the excitation efficiency of the mode, with Δ_*k*_ being a bandwidth of integration that embraces the whole resonant peak. After the integration of equation (1) the recoil force can be obtained as:


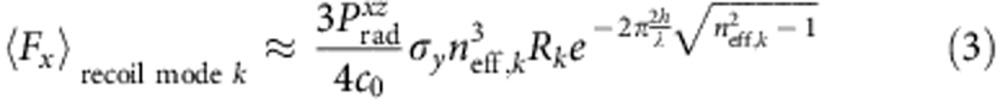


This equation carries major physical insights (green lines in Fig. 4): The force is bigger for higher mode index 
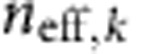
 and excitation amplitude *R*_*k*_, as expected because of the increased momentum and amplitude, respectively, of guided photonic or plasmonic modes. The distance dependence accounts for the evanescent decay of near-fields at a length 2*h*, corresponding to the ‘round-trip' of the dipolar near-fields exciting the surface mode, which in turn acts back on the dipole.

The second term in equation (2) corresponds to dipole–dipole interactions under the image-dipole quasistatic approximation. A simple expression for this term can be obtained by assuming that *r*^*p*^(*k*_t_) is equal to its quasistatic value 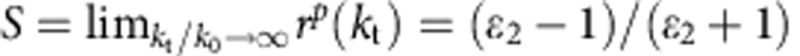, where *ɛ*_2_ is the permittivity of the first material slab at *z*=0. The approximation *r*^*p*^(*k*_t_)≈*S* corresponds to quasistatic image theory, in which every charge *q* has an associated mirror image *q*′=−*qS*. The image dipole is thus instantaneously correlated to the source by the complex amplitude *S*. This simplification makes equation (1) analytically solvable, resulting in (Supplementary Note 2):


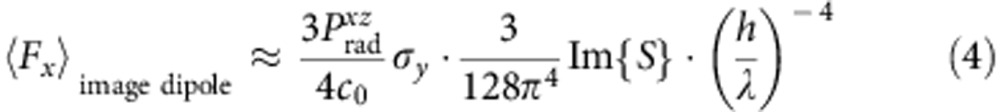


This force, having an *h*^−4^ dependence, dominates over the other components at the limit of very small distances and exists even in the absence of guided modes in the substrate or slab. The same analytical expression can also be deduced by calculating the force between two dipoles (Supplementary Note 3 and Supplementary Fig. 2) related by the image coefficient *S*, placed at a distance 
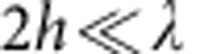
. Intriguingly, this tells us that, in general, a dipole exerts a force on another nearby dipole in a direction different to that of the line joining their centres. This finding is certainly surprising given the two-point nature of such geometry. For the lateral force to exist, the condition |*σ*_*y*_|>0 needs to be satisfied, that is, the dipoles must have a chirality in their polarization to break the *x*-mirror symmetry. In addition, the term Im{*S*} means that only those dipole components which are arg{*S*}=*π*/2 out of phase with the other dipole contribute to this force, that is, the polarization of the image dipole must lag behind to produce a lateral force. This means that a lateral force between a dipole and its image necessarily requires a lossy substrate to provide the required phase difference. In lossless materials, the image dipole is always in phase arg{*S*}=0 or anti-phase arg{*S*}=*π* to the source dipole, and the lateral force vanishes. This is in stark contrast to the vertical attractive or repulsive force from a substrate due to the image dipole^15^,^50^, which depends on Re{*S*}. Those two very particular conditions (circular polarization and losses) contribute to the elusive nature of this force.

Finally, the third term in equation (2) is the force caused by the reflected propagating components of the dipole field, given by the integral in equation (1) with its limits modified to *k*_tr_∈[0, 1], thus neglecting the evanescent components (*k*_tr_>1) of the fields. In the proximity of the substrate, this term is overshadowed by the other two terms (Fig. 4).

### Lateral force over metallic and dielectric materials

We now provide two examples to illustrate the lateral forces. First, the very general scenario of a circular dipole over a dielectric slab (in this case, silicon, *n*=3.45) on top of an infinite substrate (silica *n*=1.45), valid for any wavelength *λ* for which the dielectric is lossless (Fig. 3). The thickness of the slab *t*=0.135*λ* was optimized to achieve a maximum force (Supplementary Note 4 and Supplementary Fig. 3). Figure 2a depicts the lateral force acting on the dipole 
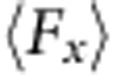
 per unit radiated power 
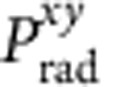
. Since we assume that the dielectric slab is lossless, the image-dipole contribution to the force (equation (4)) vanishes. The slab however supports two transverse magnetic guided modes (*n*_eff,1_=1.780, *R*_1_=0.822; *n*_eff,2_=1.072, *R*_2_=0.286) which will be unidirectionally excited by the dipole, and will exert a recoil force on the particle according to equation (3). Each mode contributes separately to the lateral force: Mode 1 decays faster with distance because of its higher effective index, but exerts a greater force as *h*→0 due to the higher momentum and amplitude of excitation.

Our second example is a circularly polarized dipole above a lossy gold substrate, modelled with a relative permittivity *ɛ*_2_=−11.8+1.28*i* at the wavelength of *λ*=632.8 nm. Such a metal surface sustains the unidirectional excitation of a surface plasmon polariton mode (*n*_eff,SPP_=1.045, *R*_SPP_=0.596) with the associated lateral recoil force (equation (3)) that exponentially decays away from the surface (Fig. 4b). Close to the surface, the main contribution to the force comes in this case from the image-dipole contribution. Owing to the imaginary part of the Au permittivity, the image dipole has the required phase lag with respect to the source dipole and exerts a lateral force on it with an *h*^−4^ distance dependence (equation (4)). To cross-check the results, Fig. 4b includes also the force obtained by evaluating numerically the spatial derivatives of the electric field using Green's function formalism^55^, showing an exact correspondence with equation (1). The time-domain numerical simulations of the fields, and a subsequent calculation of the force by integrating the Maxwell's stress tensor in a box around the dipole, also provides excellent agreement (Supplementary Note 5 and Supplementary Fig. 4).

Both examples illustrate the wide applicability of such lateral forces. To evaluate the order of magnitude of the force in experimental situations, the calculated normalized force 
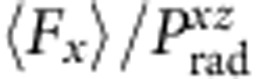
 has to be multiplied by the power radiated by the dipole 
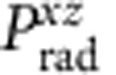
, which can be a quantum dot or an optical, microwave or radio frequency antenna. Alternatively we can consider a simple optical experiment as in Fig. 2: a polarizable nanoparticle (isotropic polarizability *α*) being illuminated by an external light source, so that it gets polarized with a polarization **p**=*α***E**_inc_ and re-radiates part of the incident power as dipolar scattering. The illuminating beam does not contribute to the lateral force, but is required to polarize the particle. In Fig. 2, the incident polarization was chosen as a diagonally linearly polarized incident plane wave. After reflection on the substrate at 45° incidence, the incident and reflected wave create a standing wave that results in an elliptically polarized field with spin components parallel to the surface at the location of the particle, as desired. We extracted the polarization **p** directly from the simulation by looking at the polarization of the scattered electric field. The resulting polarization **p** has a spin with both 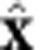 and 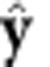 components, so we expect forces parallel to the substrate. By substituting the height-dependent polarization **p** of the particle into our analytical equation (1), we can calculate the force expected to act on the dipole equivalent to the scattering particle, and the result is shown in the dashed lines of Fig. 2. The agreement with the numerically calculated forces using Maxwell's stress tensor is very good considering the limitations of the approximations involved. The forces in Fig. 2 are normalized to an incident power density of 1 W cm^−2^, but incident power densities up to four orders of magnitude higher or more can be achieved in experiments by beam focusing. Most importantly, for small *h*, the lateral force has the same order of magnitude as the pressure force, which is easily measurable. This, together with the fact that the lateral force acts orthogonally to all other forces, and that it can be switched with the polarization, makes it susceptible to a relatively easy experimental discrimination.

## Discussion

We have shown that the scattering of a centrosymmetric, non-chiral particle over a substrate can be accompanied by a novel lateral force under polarized illumination, even if neither the particle nor the surface break the left–right symmetry. The polarization itself breaks the symmetry owing to spin–orbit coupling, allowing a precise and broadband optical control of the force direction and magnitude. The magnitude of such force is comparable with other acting electromagnetic forces.

It is worth noting that small helicity-dependent lateral forces acting on particles, similar to those described above, can arise from the transverse optical momentum and spin that exist in evanescent waves^29^ (recently measured^30^) or in interfering polarized plane waves^31^. These forces are related to unconventional spin and momentum properties of the illuminating light itself (with no need to account for back scattering), however they do not exist for small particles in the dipolar limit. They only act on particles with higher order scattering (larger particles with radii *R* such that *kR*∼1; refs 29, 30, 31). In contrast, in the present work, we described lateral forces that exist in the dipole approximation for arbitrarily small scatterers or emitters (such as subwavelength nanoparticles or atoms). For these dipolar scatterers, polarization-dependent lateral forces do not arise from a special momentum of the illumination, but instead are related to the asymmetry of the back-scattered fields that act on the particle. Studies of optical forces on small particles usually ignore the multiple scattering between the particles and surrounding environment, overlooking the complex forces that arise.

The proposed effect is not limited to a particular realization, because it involves the lateral force on a polarized dipole, which can model a variety of scenarios throughout the electromagnetic spectrum, from scattering of nanoparticles illuminated with polarized light^48^ to circularly polarized RF antennas. It is obvious that a lateral force will also exist in non-planar geometries, such as dipoles near a cylindrical waveguide^43^,^47^, and in general any geometry where spin–orbit interaction can be exploited^37^,^46^,^56^ to achieve spin-controlled recoil forces or other forms of directional scattering from structures^57^. The possible implications of a controllable lateral force existing between a dipole and a nearby surface (or between two dipoles) span from microfluidics to optomechanics, reconfigurable antennas and metamaterials.

During the review process, two related works describing the same phenomenon have come to our attention^58^,^59^, developed in parallel to ours^60^,^61^. These works strongly complement each other owing to their different viewpoints. In ref. 58, the lateral force is explained in terms of the vortex field scattered from a particle under circularly polarized illumination^62^,^63^,^64^. This vortex carries orbital angular momentum and can induce a lateral force when the symmetry of the vortex is broken by a nearby interface. An identical equation for the force (equation (1)) is obtained^58^. This explanation does not require the existence of guided modes at the interface, and hence is probably an alternative interpretation of the image-dipole force described above. Reference 58 does not explicitly explore the possibility of the recoil force arising from directional guided modes excited in the surface, which was the main motivation of our study. In contrast, ref. 59 does describe recoil forces on an emitter near a cylindrical waveguide (instead of a planar surface) exciting directional modes, and it is studied from the point of view of quantum emitters. These results reinforce our predictions that polarization-dependent lateral forces should occur for any kind of emitter or scatterer near any structure, waveguide or surface in which spin–orbit interaction results in the generation of an unbalanced electromagnetic momentum.

## Additional information

**How to cite this article:** Rodríguez-Fortuño, F. J. *et al.* Lateral forces on circularly polarizable particles near a surface. *Nat. Commun*. 6:8799 doi: 10.1038/ncomms9799 (2015).

## Supplementary Material

Supplementary Information Supplementary Figures 1-4, Supplementary Notes 1-6 and Supplementary References.
