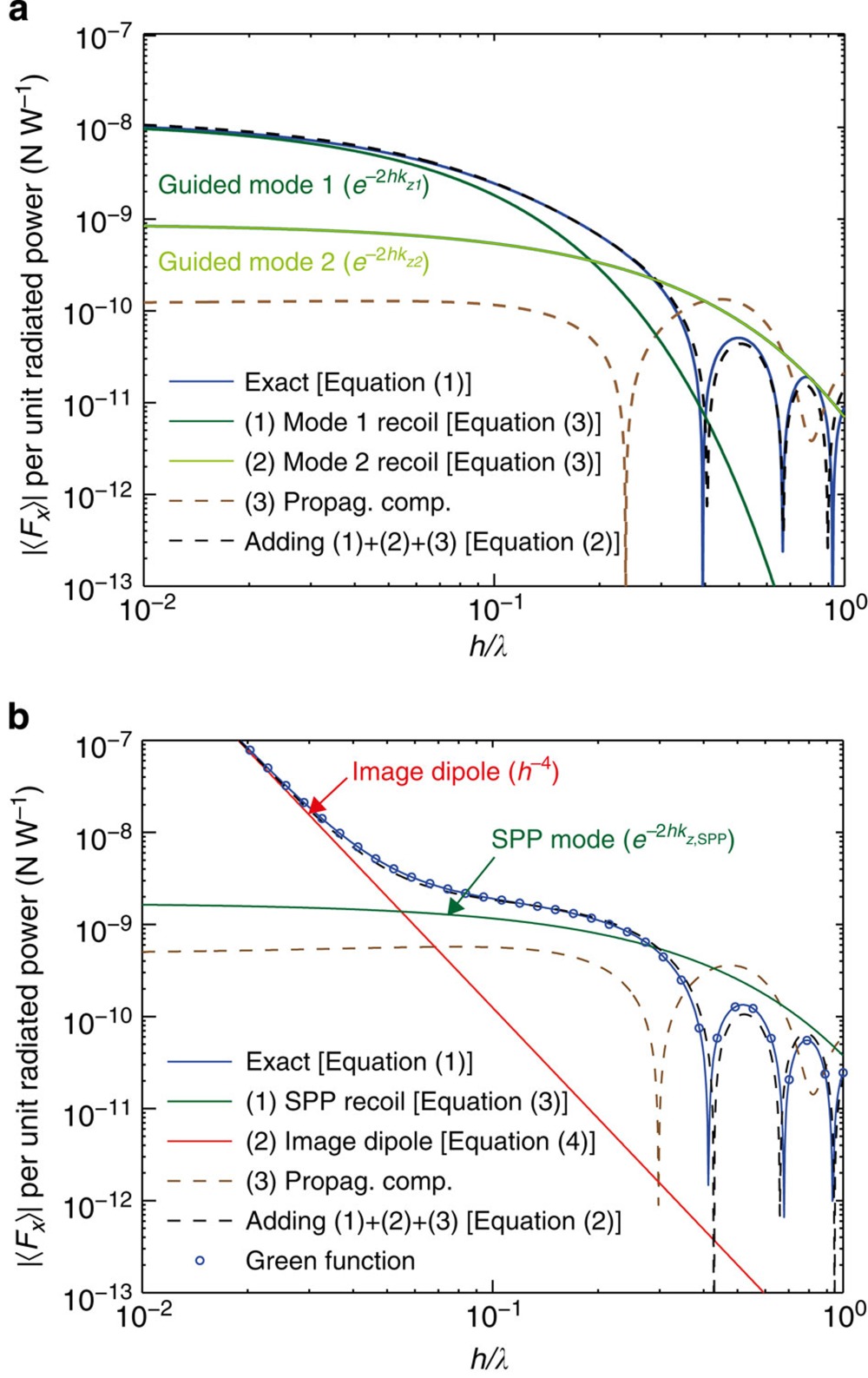# Erratum: Lateral forces on circularly polarizable particles near a surface

**DOI:** 10.1038/ncomms10263

**Published:** 2015-12-14

**Authors:** Francisco J. Rodríguez-Fortuño, Nader Engheta, Alejandro Martínez, Anatoly V. Zayats

Nature Communications 6: Article number: 879910.1038/ncomms9799 (2015); Published 11192015; Updated 12142015

Due to errors in the production process data were missing in the original version of Fig. 4b. The correct version of this figure appears below.

**Figure 4**