# Supplementary material for: Lateral forces on circularly polarizable particles near a surface
Source: Nat Commun. 2015 Nov 19;6:8799. doi: 10.1038/ncomms9799 (PMC4673490; doi:10.1038/ncomms9799)
Supplement: Supplementary Information — Supplementary Figures 1-4, Supplementary Notes 1-6 and Supplementary References. [file ncomms9799-s1.pdf]

Supplementary Figures

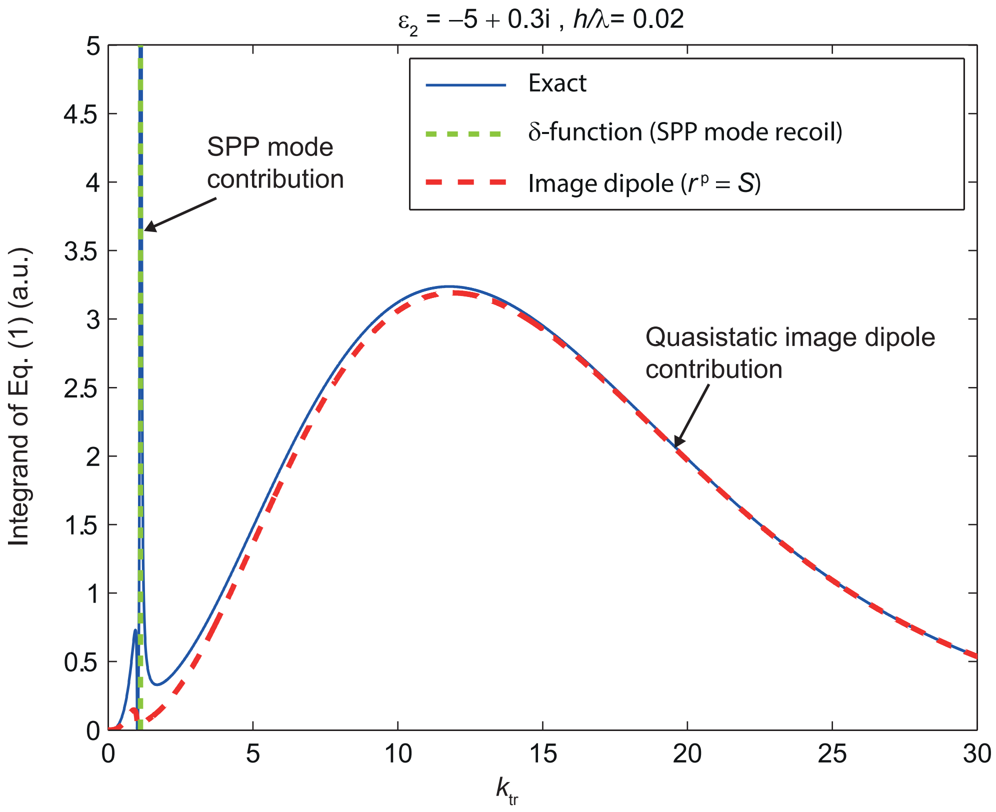

Supplementary Figure 1. Integrand of eq. (20) for a single metal substrate with $\varepsilon_2 = -5 + 0.3i$, supporting an SPP mode and an out of phase image dipole. In this case the dipole is placed at a distance $h = 0.02\lambda$. The plot of the quasistatic image dipole contribution corresponds to the integrand of eq. (20) under the approximation $r^p \approx S$, which holds for $k_{tr} \gg 1$.

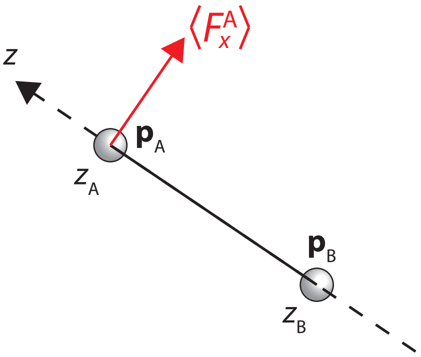

Supplementary Figure 2. Depiction of the lateral force acting between two ideal point dipoles.

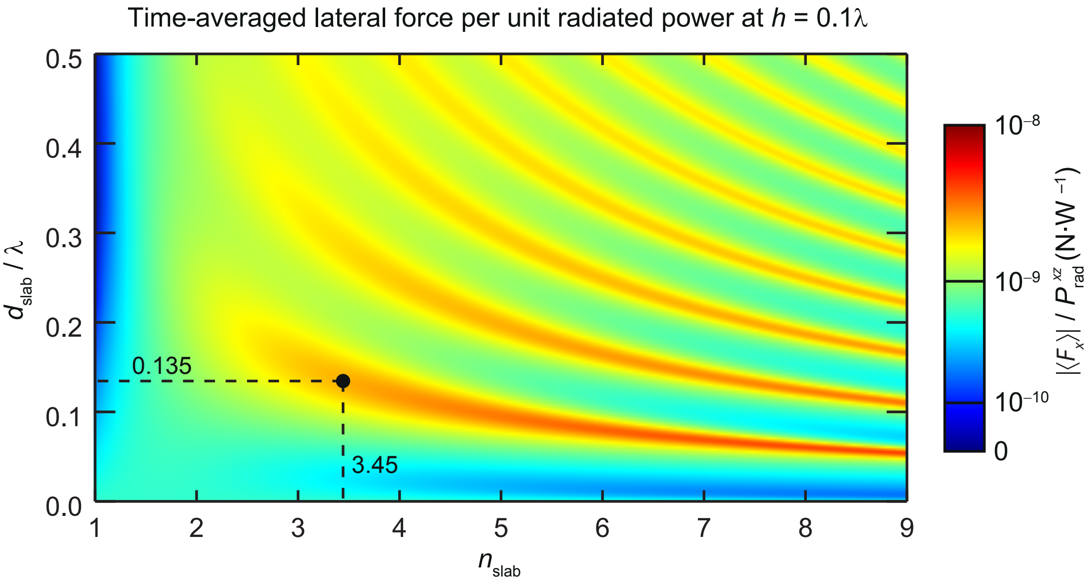

Supplementary Figure 3. Numerical calculation of the lateral force acting on a dipole over a dielectric slab. Fixed parameters considered for this calculation were $h = 0.1\lambda$, $\sigma_y = 1$, and a silica substrate $n_{subs} = 1.45$.

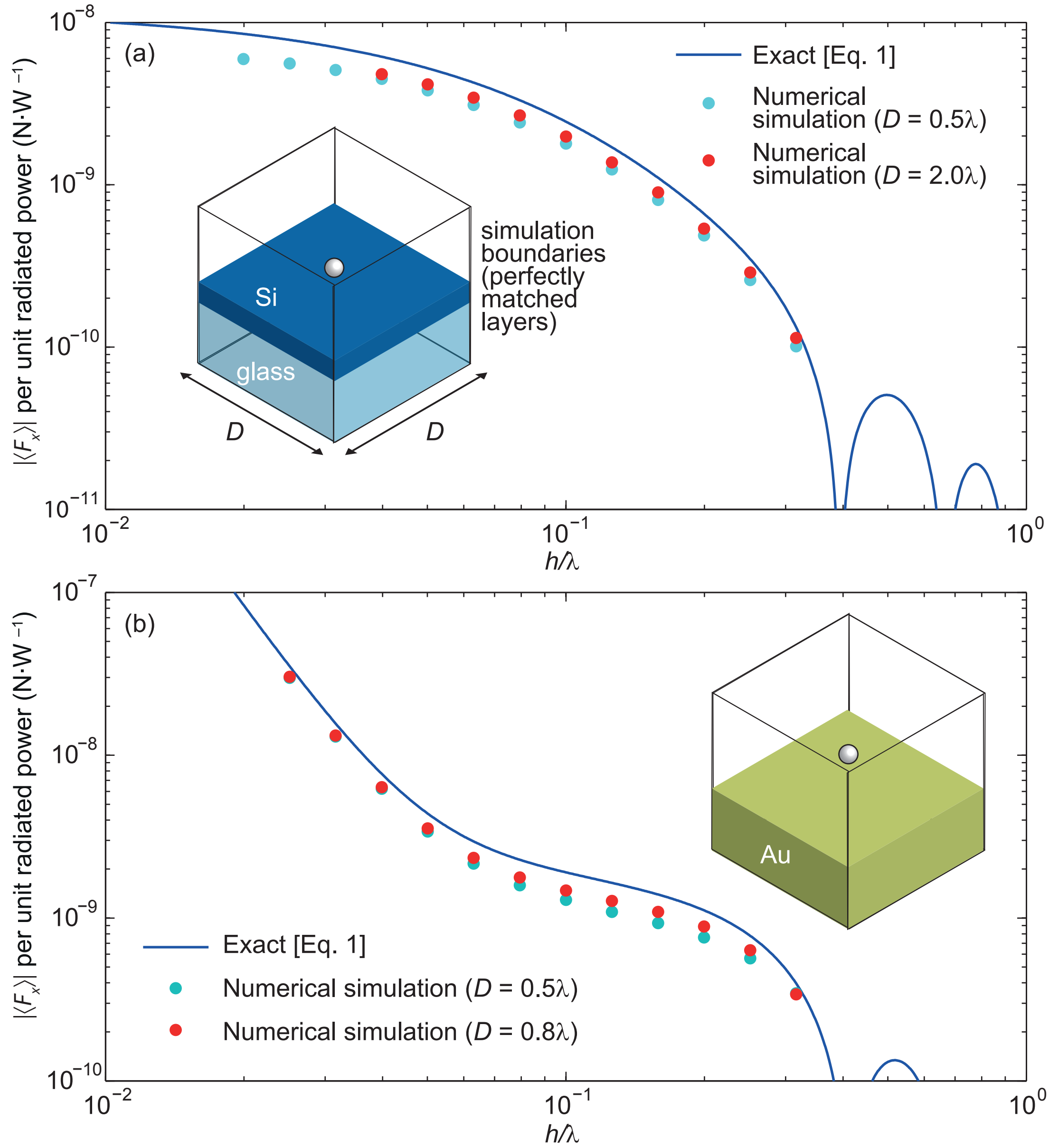

Supplementary Figure 4. Numerical calculation of the lateral force acting on a dipole using the Maxwell's stress tensor for a dipole over (a) a dielectric slab, or (b) a metal substrate, corresponding to the same parameters used by the two examples in the main text. Simulations with different simulation region sizes are included, showing that the numerical force approaches the exact analytical expression as the simulation size is increased.

Supplementary Notes

Supplementary Note 1: Derivation of the exact equation for the lateral force

Consider a dipole $\mathbf{p} = [p_x, p_y, p_z]^T$ at a distance h above a planar surface (plane $z = 0$). We will consider the general case in which the upper medium has a relative permittivity ε_1 and relative permeability μ_1, while in the main text both values are taken as 1 corresponding to free space. The surface can be a semi-infinite substrate, or any number of stacked slabs, but from the point of view of the dipole, the surface is *entirely characterised* by the Fresnel reflection coefficients $r^p(k_t)$ and $r^s(k_t)$ for p-polarized and s-polarized fields, respectively, where $\mathbf{k}_t = k_x\hat{\mathbf{x}} + k_x\hat{\mathbf{y}}$ is the transverse wave-vector associated with a given plane wave. Values of $|k_t| > k_1$ represent evanescent components, where $k_1 = n_1 k_0 = (\varepsilon_1\mu_1)^{1/2}k_0$ and $k_0 = 2\pi/\lambda$. In this section we will derive the time-averaged lateral component of the force acting on a dipole due to its own fields reflected by the surface.

The electromagnetic force acting on an ideal dipole due to external fields $\mathbf{E}$ and $\mathbf{B}$ can be calculated from first principles using the Lorentz electromagnetic force acting on each of the two point charges $\mathbf{F} = q(\mathbf{E} + \mathbf{v} \times \mathbf{B})$ that constitute the dipole, and written in terms of the spatial derivatives of the electric field at the location of the dipole. For time-harmonic fields of angular frequency ω, the time-averaged electromagnetic force on the dipole is given as (see Ref. [1]):

$$\langle \mathbf{F} \rangle = \frac{1}{2}\,\mathrm{Re}\left[p_x^* \nabla E_x(\mathbf{r}_{\mathrm{dip}}) + p_y^* \nabla E_y(\mathbf{r}_{\mathrm{dip}}) + p_z^* \nabla E_z(\mathbf{r}_{\mathrm{dip}})\right], \tag{1}$$

where the only approximation made was that the dipole is moving slowly compared to the speed of light, and $\nabla = [\partial/\partial x, \partial/\partial y, \partial/\partial z]^T$ is the gradient operator acting on the different components of the electric field $\mathbf{E}(\mathbf{r}) = [E_x, E_y, E_z]^T$ evaluated at the location of the dipole $\mathbf{r}_{\mathrm{dip}} = [0, 0, h]^T$. This electric field corresponds to that applied on the dipole, not including the self-fields of the dipole. In our scenario, $\mathbf{E}(\mathbf{r})$ corresponds to the fields reflected by the surface from the dipole back into itself.

If we are interested in the lateral component of the force, we can focus on the x-component $\langle F_x \rangle$ without loss of generality, since the x-axis can be reoriented into any desired direction within the x-y plane. From eq. (1) we can write the x-component of the force as:

$$\langle F_x \rangle = \frac{1}{2}\,\mathrm{Re}\left[p_x^* \frac{\partial E_x(x,y,z)}{\partial x}\bigg|_{\substack{x,y=0\\ z=h}} + p_y^* \frac{\partial E_y(x,y,z)}{\partial x}\bigg|_{\substack{x,y=0\\ z=h}} + p_z^* \frac{\partial E_z(x,y,z)}{\partial x}\bigg|_{\substack{x,y=0\\ z=h}}\right], \tag{2}$$

The key idea used in our work is to make use of the spatial decomposition of the electric field, such that it is written as a sum of its plane wave and evanescent components (spatial spectrum):

$$\mathbf{E}(x,y,z) = \iint_{-\infty}^{+\infty} \mathbf{E}(k_x, k_y, z) \cdot e^{ik_x x + ik_y y} \cdot dk_x dk_y, \tag{3}$$

which can be substituted into eq. (2), moving the integrals out of the derivatives, and yielding the spatial-spectrum decomposition of the lateral force:

$$\langle F_x \rangle = \iint_{-\infty}^{+\infty} \underbrace{\frac{1}{2}\,\mathrm{Re}\left[ik_x p_x^* E_x(k_x,k_y,h) + ik_x p_y^* E_y(k_x,k_y,h) + ik_x p_z^* E_z(k_x,k_y,h)\right]}_{\langle F_x(k_x,k_y)\rangle = (1/2)\,\mathrm{Re}[ik_x(\mathbf{p}^*\cdot\mathbf{E}(k_x,k_y,h))]} dk_x dk_y, \tag{4}$$

where $\left\langle F_x(k_x,k_y)\right\rangle$ represents the force acting on the dipole caused by each plane wave component of the field, which corresponds to the spatial transform of eq. (2), where the spatial derivatives $\partial/\partial x$ change to multiplication by ik_x. Notice that, although we have been speaking of decomposition into plane waves, the formalism also includes the evanescent waves of the field, which mathematically can be seen as planewaves with a transverse wave-vector greater than the wave-vector in the medium $k_t > k_1$.

Now we only have to substitute the incident fields (reflected by the substrate) into eq. (4). The analytical form of the reflected fields is developed in Supplementary Note 6, and the fields are given in eqs. (31), (32), (33a) and (33b).

Symmetry simplifications using parity

For further simplification, notice that eq. (4) has an integration over k_x and k_y from $-\infty$ to $+\infty$, so that any odd components (odd in k_x or odd in k_y) of $\left\langle F_x(k_x,k_y)\right\rangle$ will cancel out when performing the integration. Therefore, only the components of $\langle F_x\rangle$ that are *even* with both k_x and k_y will survive the integration, and will be the only ones in which we are interested.

$$\langle F_x\rangle = \iint_{-\infty}^{+\infty} \left\langle F_x(k_x,k_y)\right\rangle dk_x dk_y = \iint_{-\infty}^{+\infty} \left\langle F_x(k_x,k_y)\right\rangle\Big|_{\substack{k_x \text{ even}\\ k_y \text{ even}}} dk_x dk_y, \tag{5}$$

By looking at eq. (4), we can easily deduce that only the components of the field $\mathbf{E}(k_x,k_y,h)$ that are even in k_y *and* odd in k_x will contribute to the force.

$$\left\langle F_x(k_x,k_y)\right\rangle\Big|_{\substack{k_x \text{ even}\\ k_y \text{ even}}} = \frac{1}{2}\,\mathrm{Re}\left[ik_x\left(\mathbf{p}^*\cdot\mathbf{E}(k_x,k_y,h)\Big|_{\substack{k_x \text{ odd}\\ k_y \text{ even}}}\right)\right] \tag{6}$$

Thus, we can select and keep only the terms from the reflected field [eqs. (32), (33a) and (33b)] that show the appropriate parity in k_x and k_y, which gives us the following reflected field:

$$\mathbf{E}_{\text{ref}}(k_x,k_y,z)\Big|_{\substack{k_x \text{ odd}\\ k_y \text{ even}}} = \frac{i\omega^2\mu_0\mu_1}{8\pi^2}\left(\overleftrightarrow{\mathbf{M}}^{\mathbf{s}}_{\text{ref}}(k_x,k_y)\Big|_{\substack{k_x \text{ odd}\\ k_y \text{ even}}} + \overleftrightarrow{\mathbf{M}}^{\mathbf{p}}_{\text{ref}}(k_x,k_y)\Big|_{\substack{k_x \text{ odd}\\ k_y \text{ even}}}\right)\cdot\mathbf{p}\cdot e^{ik_{z_1}(z+h)}, \tag{7}$$

where the relevant components of the matrices are given by:

$$\begin{aligned}
\overleftrightarrow{\mathbf{M}}^{\mathbf{s}}_{\text{ref}}(k_x,k_y))\Big|_{\substack{k_x \text{ odd}\\ k_y \text{ even}}} &= 0\\
\overleftrightarrow{\mathbf{M}}^{\mathbf{p}}_{\text{ref}}(k_x,k_y))\Big|_{\substack{k_x \text{ odd}\\ k_y \text{ even}}} &= \frac{-k_x r^p(k_x,k_y)}{k_1^2}\begin{pmatrix}0 & 0 & 1\\ 0 & 0 & 0\\ -1 & 0 & 0\end{pmatrix}.
\end{aligned} \tag{8}$$

At this point we can already see why the s-polarized components of the reflected field do not affect the lateral force: because they do not have the appropriate parity in their plane wave decomposition to achieve an unbalanced force after the summation or integration of the individual forces of each of the plane wave components. Exactly the same can be said about the y-component of the source dipole $\mathbf{p}$. With such a big simplification, we may substitute eq. (8) into eq. (7), so that the relevant components of the reflected fields are given by:

$$\mathbf{E}_{\text{ref}}(k_x, k_y, z)\Big|_{\substack{k_x\ \text{odd}\\ k_y\ \text{even}}} = \frac{i\omega^2\mu_0\mu_1}{8\pi^2}\cdot\frac{k_x r^p(k_x,k_y)}{k_1^2}\cdot\begin{pmatrix}-p_z\\0\\p_x\end{pmatrix}\cdot e^{ik_{z_1}(z+h)}. \tag{9}$$

where $k_{z_1} = (k_1^2 - k_x^2 - k_y^2)^{1/2}$.

This reflected field can be substituted into the expression of the force [eqs. (5) and (6)], yielding an exact expression for the force:

$$\langle F_x\rangle = \iint_{-\infty}^{+\infty}\frac{1}{2}\,\mathrm{Re}\left[k_x^2\cdot\frac{\omega^2\mu_0\mu_1}{8\pi^2}\frac{r^p(k_x,k_y)}{k_1^2}\left(p_x^*p_z - p_z^*p_x\right)\cdot e^{ik_{z_1}(2h)}\right]dk_x dk_y, \tag{10}$$

We can work with this expression in order to simplify it

$$\begin{aligned}
\langle F_x\rangle &= \iint_{-\infty}^{+\infty}\frac{1}{2}\,\mathrm{Re}\left[k_x^2\cdot\frac{\omega^2\mu_0\mu_1}{8\pi^2}\frac{r^p(k_x,k_y)}{k_1^2}\left(p_x^*p_z - p_z^*p_x\right)\cdot e^{ik_{z_1}(2h)}\right]dk_x dk_y, && \text{Apply } \frac{\omega^2\mu_0\mu_1}{k_1^2} = \frac{1}{\varepsilon_0\varepsilon_1}.\\
&= \iint_{-\infty}^{+\infty}\frac{1}{2}\,\mathrm{Re}\left[k_x^2\cdot\frac{r^p(k_x,k_y)}{8\pi^2\varepsilon_0\varepsilon_1}\left(p_x^*p_z - p_z^*p_x\right)\cdot e^{ik_{z_1}(2h)}\right]dk_x dk_y, && \text{Apply } [p_x^*p_z - p_z^*p_x] = 2i\,\mathrm{Im}[p_x^*p_z]\\
&= \iint_{-\infty}^{+\infty}\frac{1}{2}\,\mathrm{Re}\left[2ik_x^2\cdot\frac{r^p(k_x,k_y)}{8\pi^2\varepsilon_0\varepsilon_1}\mathrm{Im}[p_x^*p_z]\cdot e^{ik_{z_1}(2h)}\right]dk_x dk_y, && \text{Apply } \mathrm{Re}(iz) = -\mathrm{Im}(z)\\
&= \iint_{-\infty}^{+\infty}-\frac{1}{2}\,\mathrm{Im}\left[2k_x^2\cdot\frac{r^p(k_x,k_y)}{8\pi^2\varepsilon_0\varepsilon_1}\mathrm{Im}[p_x^*p_z]\cdot e^{ik_{z_1}(2h)}\right]dk_x dk_y, && \text{Apply linearity}
\end{aligned} \tag{11}$$

we obtain the simplified expression:

$$\langle F_x\rangle = -\frac{1}{8\pi^2\varepsilon_0}\,\mathrm{Im}[p_x^*p_z]\iint_{-\infty}^{+\infty}k_x^2\,\mathrm{Im}\left[\frac{1}{\varepsilon_1}r^p(k_x,k_y)e^{ik_{z_1}(2h)}\right]dk_x dk_y, \tag{12}$$

now we can write the integral in terms of polar wavevector coordinates by applying the following change of variables ($k_x = k_\mathrm{t}\cos\alpha, k_y = k_\mathrm{t}\sin\alpha$), where both $r^p(k_x,k_y) = r^p(k_\mathrm{t})$ and $k_{z_1} = k_{z_1}(k_\mathrm{t})$ are independent of α, so that the integral changes according to:

$$\iint_{-\infty}^{+\infty} f(k_x,k_y)\cdot dk_x dk_y = \int_0^{+\infty}\int_0^{2\pi} f(k_\mathrm{t}\cos\alpha, k_\mathrm{t}\sin\alpha)\cdot k_\mathrm{t}\cdot d\alpha\cdot dk_\mathrm{t}, \tag{13}$$

which, applied to eq. (12) gives:

$$\langle F_x\rangle = -\frac{1}{8\pi^2\varepsilon_0}\,\mathrm{Im}[p_x^*p_z]\int_0^{+\infty}\int_0^{2\pi}k_\mathrm{t}^3\cdot\cos^2(\alpha)\,\mathrm{Im}\left[\frac{1}{\varepsilon_1}r^p(k_\mathrm{t})e^{ik_{z_1}(2h)}\right]d\alpha\cdot dk_\mathrm{t}, \tag{14}$$

since everything else is independent of α, we can evaluate the integral $\int_0^{2\pi} \cos^2(\alpha) d\alpha = \pi$, and we obtain:

$$\langle F_x \rangle = -\frac{1}{8\pi\varepsilon_0} \mathrm{Im}[p_x^* p_z] \int_0^{+\infty} k_t^3 \,\mathrm{Im}\left[\frac{1}{\varepsilon_1} r^p(k_t) e^{ik_{z1}(2h)}\right] \cdot dk_t, \tag{15}$$

Normalization of parameters

Equation (15) above constitutes an exact expression for the lateral force, and could be used as Eq. (1) in the main text (it can also be used to calculate $\left\langle F_y \right\rangle$ by rotating the axis, i.e. $p_x \rightarrow p_y$). However, working with the dipolar moments p_x and p_z is experimentally unintuitive. We would like to normalize the variables and introduce an experimentally intuitive measure of the amplitude of excitation of the dipole.

Firstly, we introduce the normalized transverse wave-vector $k_{\mathrm{tr}} = k_t/k_0$, so that $dk_t = k_0 dk_{\mathrm{tr}}$, and we can also write $k_{z1} = (k_1^2 - k_t^2)^{1/2} = k_0(n_1^2 - k_{\mathrm{tr}}^2)^{1/2}$, where $n_1 = (\varepsilon_1 \mu_1)^{1/2}$ is the effective index of medium 1, and $k_0 = 2\pi/\lambda$. Substituting all this into eq. (15) gives:

$$\langle F_x \rangle = -\frac{k_0^4}{8\pi\varepsilon_0} \mathrm{Im}[p_x^* p_z] \int_0^{+\infty} k_{\mathrm{tr}}^3 \,\mathrm{Im}\left[\frac{1}{\varepsilon_1} r^p(k_{\mathrm{tr}}) e^{i2\pi\frac{2h}{\lambda}\sqrt{n_1^2 - k_{\mathrm{tr}}^2}}\right] \cdot dk_{tr}, \tag{16}$$

We will see that it is very convenient to introduce the power radiated by the dipole as a measure of its amplitude. In order to do this, we will from now on assume that medium 1 is free space ($\varepsilon_1 = \mu_1 = n_1 = 1$). The power radiated by a dipole $\mathbf{p}$ in free space is given by:

$$\begin{aligned} P_{\mathrm{rad}} &= \frac{c_0 k_0^4}{12\pi\varepsilon_0} |\mathbf{p}|^2 \\ &= \underbrace{\frac{c_0 k_0^4}{12\pi\varepsilon_0} (|p_x|^2 + |p_z|^2)}_{P_{\mathrm{rad}}^{xz}} + \frac{c_0 k_0^4}{12\pi\varepsilon_0} |p_y|^2, \end{aligned} \tag{17}$$

so that P_{rad}^{xz} can be substituted into eq. (16) giving:

$$\langle F_x \rangle = -\frac{3}{2c_0} P_{\mathrm{rad}}^{xz} \frac{\mathrm{Im}[p_x^* p_z]}{|p_x|^2 + |p_z|^2} \int_0^{+\infty} k_{\mathrm{tr}}^3 \,\mathrm{Im}\left[r^p(k_{\mathrm{tr}}) e^{i2\pi\frac{2h}{\lambda}\sqrt{1 - k_{\mathrm{tr}}^2}}\right] \cdot dk_{\mathrm{tr}}. \tag{18}$$

Now we turn our attention to the polarization of the dipole. Let us denote the polarization vector of the dipole ignoring its y-component as $\mathbf{p}_{\mathrm{xz}} = [p_x, 0, p_z]^T$. The "spin" of a dipole polarized in the xz plane can be quantified using an expression similar to the third Stokes' parameter used to quantify the spin of a polarized electric field propagating along y.

$$\begin{aligned}
\sigma_y &= 2\frac{(\mathrm{Re}[\mathbf{p}_{xz}]\times\mathrm{Im}[\mathbf{p}_{xz}])\cdot\hat{\mathbf{y}}}{|\mathbf{p}_{\mathrm{xz}}|^2} \\
&= -2\frac{\mathrm{Im}[p_x^* p_z]}{|p_x|^2+|p_z|^2} \\
&= \frac{|p_{\mathrm{rcp}}|^2-|p_{\mathrm{lcp}}|^2}{|p_{\mathrm{rcp}}|^2+|p_{\mathrm{lcp}}|^2} = \begin{cases} +1 & \text{for clockwise (right-handed) circular polarization} \\ 0 & \text{for linear polarization} \\ -1 & \text{for anti-clockwise (left-handed) circular polarization} \end{cases}
\end{aligned} \tag{19}$$

which, upon substitution into eq. (18) yields the final expression used in the main text for the time-averaged lateral force:

$$\langle F_x\rangle = \frac{3}{4c_0}P_{\mathrm{rad}}^{xz}\sigma_y\int_0^{+\infty} k_{\mathrm{tr}}^3\,\mathrm{Im}\left[r^p(k_{\mathrm{tr}})e^{i2\pi\frac{2h}{\lambda}\sqrt{1-k_{\mathrm{tr}}^2}}\right]\cdot dk_{\mathrm{tr}}. \tag{20}$$

Supplementary Note 2: Derivation of the different terms

Motivation for the separation into terms

The final equation for the lateral force [eq. (20)] is straight forward to compute numerically, but carries a limited physical insight. By looking at the integrand (see Supplementary Fig. 1), we can understand that the force may come from several contributions.

- Firstly, we note that the reflection coefficient $r^p(k_{\mathrm{tr}})$ has a resonant behavior whenever there is a TM polarized mode supported by the surface/slab/waveguide. This will provide peaks in the integrand of eq. (20) and therefore will contribute to the force.
- On the other hand, even if r^p does not have resonances, the term $k_{\mathrm{tr}}^3\cdot\exp(4\pi h(1-k_{\mathrm{tr}}^2)^{1/2})$ will initially grow as k_{tr} grows, but will eventually decay due to the exponential term, thus a bell-shaped curve will occur in the integrand, of higher amplitude the closer the dipole is to the surface, and will also contribute to the force.

Both contributions can be clearly seen in Supplementary Fig. 1, showing a representation of the exact curve of the integrand as a function of k_{tr} for a given height and geometry, and also depicting the two contributions. Finally, when the distance h is increased into the far-field, all the components of the integrand that are above $k_{\mathrm{tr}} > 1$ vanish, together with the two terms. We are only left with the force caused by the propagating plane waves, which we define as the third term.

Mode recoil term

The reflection coefficient $r^p(k_{\mathrm{tr}})$, according to eqs. (34) and (35), exhibits resonant peaks at the values of $k_{\mathrm{tr}} = n_{\mathrm{eff},1}, n_{\mathrm{eff},2}, ...$ where $n_{\mathrm{eff,k}}$ is the effective index of the k-th TM guided mode of the surface. Since $r^p(k_{\mathrm{tr}})$ appears in the integrand of eq. (20), the peaks will contribute to the force. The higher the mode excitation, the higher this contribution will be. We interpret this as the recoil force due to the directional excitation of the guided mode. It is known that any TM mode supported by the surface will be excited directionally by a circularly polarized dipole, as discussed in the main text.

To find an approximate expression for this term, we simply approximate the imaginary part of $r^p(k_{\text{tr}})$ as a series of δ-functions corresponding to the different TM modes supported by the waveguide, labelled from $k = 1...N$ (N can be zero if there are no supported modes, so this term vanishes):

$$\text{Im}[r^p(k_{\text{tr}})] \approx \sum_{k=1}^{N} R_k \delta(k_{\text{tr}} - n_{\text{eff},k}) \tag{21}$$

where R_k is the area under the resonant peak in r^p, as defined in the main text, and $n_{\text{eff},k}$ is the effective index of the k-th mode.

Thanks to the sieving property of the δ function $\int \delta(x - x_0) f(x) dx = f(x_0)$, it is trivial to substitute eq. (21) into eq. (20) and perform the integration, resulting in Eq. 3 in the main text.

Image dipole term

For the image dipole term, we apply the quasistatic approximation to the reflection coefficient. In the quasistatic limit, the z-component of the wave-vector is given by $k_{zi} = (n_i k_0^2 - k_{\text{tr}}^2)^{1/2} \xrightarrow{k_{\text{tr}}>>k_0} k_{zi} \approx i k_{\text{tr}}$, which when substituted into the equations of the reflection coefficient [eqs. (34) and (35)] result in a constant value for r^p. Therefore, we can take r^p to be constant and equal to the complex image coefficient S:

$$r^p(k_{tr}) \approx S = \lim_{k_{tr}\to\infty} r^p(k_{\text{tr}}) = \frac{\varepsilon_2 - \varepsilon_1}{\varepsilon_2 + \varepsilon_1} \tag{22}$$

where ε_1 and ε_2 are the relative permittivities of the medium above and below the $z = 0$ surface, respectively. If r^p is approximated by the constant S, it can be taken out of the integral in eq. (20), yielding:

$$\langle F_x \rangle = \frac{3}{4c_0} P_{\text{rad}}^{xz} \sigma_y \, \text{Im}\left[S \int_0^{+\infty} k_{\text{tr}}^3 e^{i2\pi \frac{2h}{\lambda} \sqrt{1-k_{\text{tr}}^2}} \cdot dk_{\text{tr}} \right]. \tag{23}$$

and the integral now has an analytical solution, as follows:

$$\begin{aligned} &\text{Im}\left[S \int_0^{+\infty} k_{\text{tr}}^3 e^{i2\pi \frac{2h}{\lambda} \sqrt{1-k_{\text{tr}}^2}} \cdot dk_{\text{tr}} \right] \\ &= \frac{3\,\text{Im}[S] \cos(4\pi h/\lambda)}{128\pi^4} \left(\frac{h}{\lambda}\right)^{-4} + \frac{3\,\text{Im}[S] \sin(4\pi h/\lambda)}{32\pi^3} \left(\frac{h}{\lambda}\right)^{-3} - \frac{\text{Im}[S] \cos(4\pi h/\lambda)}{8\pi^2} \left(\frac{h}{\lambda}\right)^{-2} \\ &+ \frac{3\,\text{Re}[S] \sin(4\pi h/\lambda)}{128\pi^4} \left(\frac{h}{\lambda}\right)^{-4} - \frac{3\,\text{Re}[S] \cos(4\pi h/\lambda)}{32\pi^3} \left(\frac{h}{\lambda}\right)^{-3} - \frac{\text{Re}[S] \sin(4\pi h/\lambda)}{8\pi^2} \left(\frac{h}{\lambda}\right)^{-2}. \end{aligned} \tag{24}$$

The quasistatic approximation is only valid when the electric dipole source is very close to the surface, in the limit $h \to 0$, therefore we can make a Taylor expansion around $h = 0$ of the sine and cosine functions in eq. (24) and keep only the terms with the most negative exponent of h, which will dominate over the others at low heights. This gives:

$$\text{Im}\left[S \int_0^{+\infty} k_{\text{tr}}^3 e^{i4\pi \frac{h}{\lambda} \sqrt{1-k_{\text{tr}}^2}} \cdot dk_{\text{tr}} \right] \approx \frac{3\,\text{Im}[S]}{128\pi^4} \left(\frac{h}{\lambda}\right)^{-4} + \text{higher order terms...} \tag{25}$$

Substituting eq. (25) into eq. (23) we arrive at:

$$\langle F_x \rangle \approx \frac{3}{4c_0} P^{xz}_{\text{rad}} \sigma_y \frac{3}{128\pi^4} \operatorname{Im}[S] \left(\frac{h}{\lambda}\right)^{-4}. \tag{26}$$

which is the final form of Eq. (4) in the main text.

Also notice that, by assuming $r^p \approx S$, the reflected fields of the substrate [eqs. (32), (33a) and (33b)] are exactly equivalent to the fields created by a dipole [eqs. (28), (29), (30a) and (30b)] whose dipole moment corresponds to associating each charge q of the source dipole to a mirrored image charge $q' = -Sq$, resulting in an image dipole with polarization $\mathbf{p}_{\text{image}} = [-Sp_x, -Sp_y, Sp_z]$ located at $z = -h$. This is consistent with image-theory of static charges over a substrate. For this reason, this term represents the contribution to the force of the quasistatic image dipole.

Supplementary Note 3. Lateral force between two arbitrary dipoles

We can generalise the previous result to the case of two arbitrarily polarized dipoles. Consider two dipoles with polarization $\mathbf{p}_A$ and $\mathbf{p}_B$ separated a distance d. Without loss of generality, we can assume dipole A to be located at $(x, y, z) = (0, 0, z_A)$ and dipole B at $(x, y, z) = (0, 0, z_B)$, as shown in Supplementary Fig. 2. We can calculate the force produced by dipole B on dipole A. The fields of dipole B can be obtained by using eqs. (28), (29), (30a) and (30b), and substituted into the expression of the force eq. (1). Following the same steps we did for the dipole over the substrate, we arrive at a final expression for the lateral force (x component) exerted between the two dipoles:

$$\left\langle F^A_x \right\rangle = \mp \frac{3}{4c_0} \sqrt{P^A_{\text{rad},xz} P^B_{\text{rad},xz}} \cdot \operatorname{Re}\left[\frac{p^{A*}_x p^B_z + p^{A*}_z p^B_x}{|\mathbf{p}^A_{xz}| \cdot |\mathbf{p}^B_{xz}|} \cdot \int_0^{+\infty} k^3_{\text{tr}} e^{ik_z |z_A - z_B|} \cdot dk_{\text{tr}} \right]. \tag{27}$$

where the upper sign is used if $z_A > z_B$ and viceversa, and $\mathbf{p}^{A,B}_{xz}$ corresponds to the dipole moment of dipoles A or B neglecting the y-component. It is surprising that such a two point system can actually exhibit a lateral force between the dipoles, orthogonal to the line joining them, thanks to their polarization.

If we make dipole B equal to an image dipole $\mathbf{p}_B = -S \cdot [p^A_x, p^A_y, -p^A_z]$, as quasistatic image theory requires, then we have:

$$\begin{aligned} \sqrt{P^A_{\text{rad},xz} P^B_{\text{rad},xz}} &= |S| P^A_{\text{rad},xz}, \\ |\mathbf{p}^A_{xz}| \cdot |\mathbf{p}^B_{xz}| &= |S| |\mathbf{p}^A_{xz}|^2, \text{and} \\ p^{A*}_x p^B_z + p^{A*}_z p^B_x &= S(p^{A*}_x p^A_z - p^{A*}_z p^A_x) = 2iS \operatorname{Im}[p^{A*}_x p^A_z] = -iS \cdot \sigma_y \cdot |\mathbf{p}^A_{xz}|^2, \end{aligned}$$

which makes eq. (27) identical to eq. (23), as expected.

Supplementary Note 4: Optimization of dielectric slab

Example 1 in the main text considers a dielectric slab with index n_{slab} and thickness t_{slab} placed on top of an infinite substrate with index n_{subs}. Such a structure will support guided TM modes in the slab, and will be suitable for achieving lateral forces on nearby circularly polarized dipoles.

The index (n_{slab}) and thickness (t_{slab}) of the slab will determine the existence of TM modes, as well as their effective index and excitation amplitudes, and therefore the recoil forces. In this section, we perform a numerical study in order to optimize the thickness of the slab, considering a substrate of $n_{\mathrm{subs}} = 1.45$. We numerically apply eqs. (20) and (35) for different values of n_{slab} and t_{slab}. The resulting force map at $h = 0.1\lambda$, valid at any frequency, is shown in Supplementary Fig. 3.

Supplementary Note 5: Numerical simulations

In order to cross-check the results presented in the main text, we performed a time-domain numerical calculation of the electromagnetic fields and the associated optical forces in the two scenarios described in the main text. We used the commercial simulation software CST Microwave Studio. Results are shown in Supplementary Fig. 4.

We simulated the circularly polarized point dipole by using two orthogonal subwavelength discrete current ports, excited $\pi/2$ out of phase to each other, placed at a variable distance h above a surface. The boundary conditions in all directions were set to perfectly matched layers (PMLs). The surface at $z = 0$ was a dielectric slab over a dielectric substrate, in the first example, or a metallic substrate, in the second example, as described in the main text.

After obtaining the electromagnetic fields created by the dipole, we computed Maxwell's stress tensor in the space surrounding the dipole, and integrated it in a box surrounding the dipole, to obtain the total average force acting on it [1]. We repeated this procedure for different values of the distance h. To make sure that our simulation results of the force were consistent, we made sure that changing the size of the box of integration resulted in the same value of the force, since the force calculated by integrating Maxwell's stress tensor on a box depends only on the objects contained within it, and not on the size or shape of the box [1].

Also, we noticed that the size of the simulation region affected our results. It is well known that PML boundary conditions are not perfect, and they always show some unwanted reflection. Ideally, the PML layers should be as far from the dipole as possible for best results, requiring longer simulation times. As shown in Supplementary Fig. 4, the numerical calculation of the force approaches the analytical calculation as the size of the simulation region (D) is increased.

Supplementary Note 6: Spatial decomposition of dipole fields and reflected fields

The fields of the source dipole can be written as a superposition of plane waves and evanescent waves with different transverse wave-vectors $\left(k_x, k_y\right)$ for both s-polarized and p-polarized plane waves, as:

$$\mathbf{E}_{\text{dip}}(\mathbf{r}) = \iint_{-\infty}^{+\infty} \overbrace{\mathbf{E}_{\text{dip}}(k_x, k_y) \cdot e^{ik_{z_1}|z-h|}}^{\mathbf{E}_{\text{dip}}(k_x,k_y,z)} \cdot e^{ik_x x + ik_y y} dk_x dk_y,$$
$$\text{where } \mathbf{E}_{\text{dip}}(k_x, k_y) = \underbrace{\mathbf{E}^{\mathbf{s}}_{\text{dip}}(k_x, k_y)}_{\text{s-polarized}} + \underbrace{\mathbf{E}^{\mathbf{p}}_{\text{dip}}(k_x, k_y)}_{\text{p-polarized}} \tag{28}$$

For a dipole source $\mathbf{p} = [p_x, p_y, p_z]^T$, the spatial spectrum of the electric field can be deduced by applying the Weyl mathematical identity to the well-known expression for the fields of a dipole in free space, and is given by (see Ref. [1]):

$$\mathbf{E}_{\mathbf{dip}}(k_x, k_y) = \frac{i\omega^2 \mu_0 \mu_1}{8\pi^2} \left(\overleftrightarrow{\mathbf{M}}\,\mathbf{p}\right),$$
$$\text{where } \overleftrightarrow{\mathbf{M}} = \overleftrightarrow{\mathbf{M}}^{\mathbf{s}}(k_x, k_y) + \overleftrightarrow{\mathbf{M}}^{\mathbf{p}}(k_x, k_y) \tag{29}$$

where the tensor matrices $\overleftrightarrow{\mathbf{M}}^{\mathbf{s}}$ and $\overleftrightarrow{\mathbf{M}}^{\mathbf{p}}$ are:

$$\overleftrightarrow{\mathbf{M}}^{\mathbf{s}}(k_x, k_y) = \frac{1}{k_{z_1}(k_x^2 + k_y^2)} \begin{pmatrix} k_y^2 & -k_x k_y & 0 \\ -k_x k_y & k_x^2 & 0 \\ 0 & 0 & 0 \end{pmatrix} \tag{30a}$$

$$\overleftrightarrow{\mathbf{M}}^{\mathbf{p}}(k_x, k_y) = \frac{1}{k_1^2(k_x^2 + k_y^2)} \begin{pmatrix} k_x^2 k_{z_1} & k_x k_y k_{z_1} & \mp k_x(k_x^2 + k_y^2) \\ k_x k_y k_{z_1} & k_y^2 k_{z_1} & \mp k_y(k_x^2 + k_y^2) \\ \mp k_x(k_x^2 + k_y^2) & \mp k_y(k_x^2 + k_y^2) & (k_x^2 + k_y^2)^2 / k_{z_1} \end{pmatrix}, \tag{30b}$$

and the upper sign is used for $z > h$ while the lower sign is used for $z < h$.

Knowing the fields generated by the dipole (often called the primary fields) we can now calculate the reflected fields by the surface, simply by considering that each plane wave component will be reflected according to its polarization and its transverse wavevector k_{t} with the appropriate Fresnel reflection coefficient $r^p(k_{\text{t}})$ or $r^s(k_{\text{t}})$ (mathematically this also works for the evanescent field components, which have the same analytical form as plane waves simply by taking $k_{\text{t}} > k_1$ which makes k_{z1} imaginary). Doing this yields the spatial spectrum of the reflected fields:

$$\mathbf{E}_{\text{ref}}(\mathbf{r}) = \iint_{-\infty}^{+\infty} \overbrace{\mathbf{E}_{\text{ref}}(k_x, k_y) \cdot e^{ik_{z_1}(z+h)}}^{\mathbf{E}_{\text{ref}}(k_x,k_y,z)} \cdot e^{ik_x x + ik_y y} dk_x dk_y,$$
$$\text{where } \mathbf{E}_{\text{ref}}(k_x, k_y) = \underbrace{\mathbf{E}^{\mathbf{s}}_{\text{ref}}(k_x, k_y)}_{\text{s-polarized}} + \underbrace{\mathbf{E}^{\mathbf{p}}_{\text{ref}}(k_x, k_y)}_{\text{p-polarized}} \tag{31}$$

where, similar to what we did previously

$$\mathbf{E_{ref}}(k_x, k_y) = \frac{i\omega^2\mu_0\mu_1}{8\pi^2}\left(\overleftrightarrow{\mathbf{M}}_{\mathrm{ref}}\,\mathbf{p}\right),$$
$$\text{where } \overleftrightarrow{\mathbf{M}}_{\mathrm{ref}} = \overleftrightarrow{\mathbf{M}}^{\mathbf{s}}_{\mathrm{ref}}(k_x, k_y) + \overleftrightarrow{\mathbf{M}}^{\mathbf{p}}_{\mathrm{ref}}(k_x, k_y) \tag{32}$$

and the tensors $\overleftrightarrow{\mathbf{M}}^{\mathbf{s}}_{\mathrm{ref}}(k_x, k_y)$ and $\overleftrightarrow{\mathbf{M}}^{\mathbf{p}}_{\mathrm{ref}}(k_x, k_y)$ are given by:

$$\overleftrightarrow{\mathbf{M}}^{\mathbf{s}}_{\mathrm{ref}}(k_x, k_y) = \frac{r^s(k_x, k_y)}{k_{z_1}(k_x^2 + k_y^2)}\begin{pmatrix} k_y^2 & -k_x k_y & 0 \\ -k_x k_y & k_x^2 & 0 \\ 0 & 0 & 0 \end{pmatrix} \tag{33a}$$

$$\overleftrightarrow{\mathbf{M}}^{\mathbf{p}}_{\mathrm{ref}}(k_x, k_y) = \frac{-r^p(k_x, k_y)}{k_1^2(k_x^2 + k_y^2)}\begin{pmatrix} k_x^2 k_{z_1} & k_x k_y k_{z_1} & +k_x(k_x^2 + k_y^2) \\ k_x k_y k_{z_1} & k_y^2 k_{z_1} & +k_y(k_x^2 + k_y^2) \\ -k_x(k_x^2 + k_y^2) & -k_y(k_x^2 + k_y^2) & -(k_x^2 + k_y^2)^2/k_{z_1} \end{pmatrix}. \tag{33b}$$

These reflected fields are often called the secondary fields of the dipole, and are the ones responsible of exerting a force on it. All these expressions can be found in Ref. [1].

For a single interface (upper medium with relative perittivity ε_i, and lower medium with relative permittivity ε_j), the reflection coefficient is given by:

$$r^p(k_x, k_y) = r^p(k_t) = \frac{\varepsilon_2 k_{z1} - \varepsilon_1 k_{z2}}{\varepsilon_2 k_{z1} + \varepsilon_1 k_{z2}}, \tag{34}$$

where $k_{zi} = (k_i^2 - k_{\mathrm{t}}^2)^{1/2} = k_0(n_i^2 - k_{\mathrm{tr}}^2)^{1/2}$. For a single slab of permittivity ε_2 and thickness t over a substrate of permittivity ε_3, the p-polarized reflection coefficient is given by:

$$r^p(k_x, k_y) = r^p(k_t) = \frac{r^p_{1,2} + r^p_{2,3}e^{2ik_{z2}t}}{1 + r^p_{1,2}r^p_{2,3}e^{2ik_{z2}t}}, \tag{35}$$

where $r^p_{i,j}$ correspond to a single interface between medium ε_i and ε_j taken from eq. (34).

Supplementary References

1. Novotny, L. & Hecht, B. *Principles of Nano-Optics.* (Cambridge University Press, New York, 2011).

2. Johnson, P. B. & Christy, R. W. Optical Constants of the Noble Metals. *Phys. Rev. B* **6**, 4370–4379 (1972).
